# Theoretical Analysis, Neural Network-Based Inverse Design, and Experimental Verification of Multilayer Thin-Plate Acoustic Metamaterial Unit Cells

**DOI:** 10.3390/ma19010152

**Published:** 2026-01-01

**Authors:** An Wang, Chi Cai, Ying You, Yizhe Huang, Xin Zhan, Linfeng Gao, Zhifu Zhang

**Affiliations:** 1 Hubei Key Laboratory of Modern Manufacturing Quality Engineering, School of Mechanical Engineering, Hubei University of Technology, Wuhan 430068, China; 102500010@hbut.edu.cn (A.W.); 102500042@hbut.edu.cn (C.C.); yizhehuang@hbut.edu.cn (Y.H.); 2 State Key Laboratory of Digital Manufacturing Equipment and Technology, Huazhong University of Science and Technology, Wuhan 430074, China; 3 Dongfeng Liuzhou Motor Co., Ltd., Liuzhou 545005, China; 4 School of Mechanical and Electrical Engineering, Hainan University, Haikou 570228, China

**Keywords:** multilayer composite thin-plate acoustic metamaterial, sound insulation performance, neural network, inverse design

## Abstract

Acoustic metamaterials are artificially engineered materials composed of subwavelength structural units, whose effective acoustic properties are primarily determined by structural design rather than intrinsic material composition. By introducing local resonances, these materials can exhibit unconventional acoustic behavior, enabling enhanced sound insulation beyond the limitations of conventional structures. In this study, a thin plate (thin sheet) refers to a structural element whose thickness is much smaller than its in-plane dimensions and can be accurately described using classical thin-plate vibration theory. When resonant mass blocks are attached to a thin plate, a thin-plate acoustic metamaterial is formed through the coupling between plate bending vibrations and local resonances. Thin-plate acoustic metamaterials exhibit excellent sound insulation performance in the low- and mid-frequency ranges. Multilayer configurations and the combination with porous materials can effectively broaden the insulation bandwidth and improve overall performance. However, the large number of structural parameters in multilayer composite thin-plate acoustic metamaterials significantly increases design complexity, making conventional trial-and-error approaches inefficient. To address this challenge, a neural-network-based inverse design framework is proposed for multilayer composite thin-plate acoustic metamaterials. An analytical model of thin-plate metamaterials with multiple attached cylindrical masses is established using the point matching and modal superposition methods and validated by finite element simulations. A multilayer composite unit cell is then constructed, and a dataset of 30,000 samples is generated through numerical simulations. Based on this dataset, a forward prediction network achieves a test error of 1.06%, while the inverse design network converges to an error of 2.27%. The inverse-designed structure is finally validated through impedance tube experiments. The objective of this study is to establish a systematic theoretical and neural-network-assisted inverse design framework for multilayer thin-plate acoustic metamaterials. The main novelties include the development of an accurate analytical model for thin-plate metamaterials with multiple attached masses, the construction of a large-scale simulation dataset, and the proposal of a neural-network-assisted inverse design strategy to address non-uniqueness in inverse design. The proposed approach provides an efficient and practical solution for low-frequency sound insulation design.

## 1. Introduction

Metamaterial technology offers a new approach to controlling mid- and low-frequency noise. Acoustic metamaterials overcome the physical limitations of natural materials through subwavelength structural design and exhibit unusual acoustic properties such as negative mass density and negative elastic modulus [1]. These materials can effectively break the constraints of the mass law and significantly enhance sound insulation performance in the low- and mid-frequency ranges. Thin-plate acoustic metamaterials are lightweight and have adjustable sound insulation frequencies. Multilayer structures can further broaden the insulation bandwidth and improve design flexibility. However, such designs also introduce challenges, including structural complexity and numerous design parameters. Traditional design methods depend heavily on extensive experiments and parameter scanning, resulting in low efficiency. Neural networks, with their strong learning and modeling capabilities, offer an effective solution. Applying neural networks to the design of acoustic metamaterials can greatly improve design efficiency and optimization potential. Thin-film acoustic metamaterials were first proposed by Yang et al. in 2008 [2]. This type of structure breaks the traditional mass law limitation in the low-frequency range and provides excellent sound insulation performance. It mainly consists of a mass block, an elastic film, and a supporting frame. The mass block is attached to the elastic film, forming a spring–mass system. The sound insulation band can be tuned by adjusting the parameters of the film and the mass block. Naify et al. [3] modified the single-mass-block design into a composite annular mass configuration and studied how the arrangement of multiple unit cells affects sound insulation. Xiao et al. [4] investigated a double-layer membrane acoustic metamaterial by designing a double-layer thin-film unit and adjusting the film tension through different magnetic blocks. Within the frequency range of 70–200 Hz, the structure achieved an average sound insulation level of 40 dB.

Xu et al. [5] proposed a new type of thin-film acoustic metamaterial composed of three parts: a free film, a supporting grating, and a back cavity. Both theoretical and experimental studies were conducted, showing that the material exhibited excellent sound absorption performance across a wide frequency range below 500 Hz. Lu et al. [6] introduced a split-ring mass block design, which effectively enhanced the acoustic properties by adjusting the mass block layout. Zhou et al. [7] developed a symmetrical cross-shaped mass block thin-film metamaterial with continuous multi-state anti-resonance characteristics. This structure significantly expanded the sound attenuation range in the low-frequency region and exhibited single-negative equivalent parameter behavior, resulting in multiple continuous sound insulation peaks. Zhang et al. [8] established a theoretical model for thin-film metamaterials with attached cylindrical mass blocks. The mass block was treated as an additional surface mass on the thin plate, and the Galerkin method was used to calculate the sound insulation. However, this method was limited to structures with a single mass block. Tian et al. [9] later developed a theoretical model for a thin-film metamaterial with an attached ring mass block. Chen et al. [10] applied the point matching method, converting the force exerted by the mass block on the film into a set of point forces, which improved the accuracy of the theoretical predictions. Huang et al. [11] designed a novel petal-shaped annular mass block and analyzed the influence of its split-ring parameters. Simulation and experimental results confirmed the effectiveness of the design and the accuracy of the model. The sound insulation effects and corresponding vibration modes of four different structures were examined to reveal the sound insulation mechanism. The effects of parameters such as ring width, central angle, mass position, and weight on the sound insulation characteristics were also investigated. Huang et al. [12] further integrated bionic concepts into thin-film metamaterial design. Inspired by spider web topology, two structural models were developed using polymer membranes combined with resonators. Numerical simulations and experimental validations showed that the bionic structures generated unique resonance modes between the membrane and the resonator. The results demonstrated that the design significantly broadened the sound attenuation bandwidth while reducing weight. This bionic strategy provides a new pathway for developing lightweight and high-performance sound insulation devices. Ciaburro et al. [13] constructed a metamaterial using cork film as the membrane and thumbtacks and buttons as mass blocks. The structure achieved excellent sound insulation performance in the frequency range of 200–600 Hz. Li et al. [14] proposed a semi-analytical model for calculating the sound insulation of a new thin-film metamaterial. This model can quickly evaluate the sound insulation of a rectangular membrane with arbitrary shape and surface density. Using conformal mapping theory, the shape of the mass block was transformed, simplifying the computation process. The results were verified through finite element simulations. The study showed that the surface mass density strongly affects both the magnitude and frequency distribution of the sound insulation, and that the center-of-mass position plays a significant role in acoustic behavior. Li et al. [15] combined a double-membrane acoustic metamaterial with a Helmholtz resonator to design a new “sandwich-type” metamaterial. Theoretical calculations determined the resonant frequency and displacement of the structure, while numerical simulations and experiments verified its sound insulation performance. The results confirmed that the structure achieved excellent insulation and could be flexibly designed to meet practical needs. Although thin-film acoustic metamaterials show good performance in the low- and mid-frequency ranges, they require membrane tension to function effectively. In practical applications, film materials may deform over time due to aging, causing a reduction in tension and loss of functionality. These factors limit their widespread industrial use. In contrast, thin-plate materials offer greater durability and do not rely on pre-tension, making them more suitable for industrial applications. Oudich et al. [16] proposed a new type of thin-plate acoustic metamaterial featuring single and composite rubber columns attached to the surface of a uniform thin plate.

The local resonant unit composed of rubber columns and thin plates can generate a low-frequency band gap. Adjusting the height and cross-sectional area of the rubber columns allows control of the band gap width. Experimental results confirmed the presence of a complete acoustic band gap, which agreed well with theoretical calculations. Assouar et al. [17] investigated the sound insulation performance of a thin plate with a spring–oscillator structure under different incident angles and substrate thicknesses, and compared it with that of a flat plate without an oscillator. De et al. [18] incorporated a metamaterial structure into a double-layer plate to improve its sound insulation performance. Xiao et al. [19] proposed a new acoustic metamaterial laminate composed of a carbon fiber reinforced plate and a two-degree-of-freedom spring–mass–damping system. This design effectively reduced interior noise when applied in vehicles. Ye et al. [20] combined four thin-plate metamaterial units with different attached mass blocks into a composite unit to enhance the sound insulation peak. Tan et al. [21] analyzed the insulation performance of single-, double-, and triple-layer thin-plate metamaterials. The results showed that stacking metamaterials with different thicknesses can produce multiple sound insulation peaks. Langfeldt et al. [22] studied the sound insulation properties of metamaterials with various mass blocks and multilayer structures. They employed a particle swarm optimization algorithm to optimize the 100–400 Hz frequency band. A theoretical model of a thin-plate metamaterial containing strip-shaped mass blocks was also developed to estimate the modal characteristics, effective mass, and sound insulation performance. The model was validated through finite element simulations. Lin et al. [23] designed a sandwich-type thin-plate acoustic metamaterial featuring composite resonance and established a theoretical model for its sound transmission loss. Based on this model, the effects of several key parameters on sound insulation performance were analyzed. Wang et al. [24] combined a double-layer thin-plate metamaterial with a porous material to form a composite acoustic structure. Compared with a traditional double-layer homogeneous thin plate, this composite exhibited superior sound insulation in the 208–850 Hz frequency range. Gu et al. [25] developed a multilayer composite structure consisting of thin-plate metamaterials and porous materials, and analyzed how structural parameters affect the sound insulation behavior of the composite system.

Neural networks are an essential component of artificial intelligence. With the rapid development of computer technology, artificial intelligence has drawn significant research attention and has been widely applied in fields such as machine translation, target detection, autonomous driving, speech recognition, and image classification [26]. Beyond computer science, artificial intelligence is also used in various industries, including education, healthcare, agriculture, and animal husbandry [27]. In recent years, neural-network-based metamaterial design has become a major research direction in metamaterial development. Li et al. [28] applied neural networks to the inverse design of band gaps in two-dimensional phononic crystals. By training autoencoders to extract topological features from images and using finite element simulations to obtain corresponding band gaps, they established a neural network model to learn the implicit relationship between band gap and topology. The desired band gap could then be input to predict the corresponding topological features. Zheng et al. [29] employed a Gaussian–Bayesian machine learning model for the inverse design of acoustic metamaterials. Through iterative learning, prediction cycles, and adaptive acquisition functions, the model achieved high efficiency. A typical absorber was designed and its performance was verified experimentally and numerically. Bacigalupo et al. [30] used a neural network to optimize the maximum band gap in the low-frequency range of periodic acoustic metamaterials, which greatly reduced computational effort. Zhang et al. [31] adopted a generative adversarial network (GAN) to design sound-absorbing porous materials. A dataset was generated through finite element simulations, and eight representative designs produced by the GAN were analyzed. Two of these designs were experimentally validated, confirming the network’s effectiveness. Lai et al. [32] used a conditional Wasserstein GAN to design metamaterials with minimized total scattering planes of the scatterer. Gao et al. [33] developed a one-dimensional convolutional neural network model that included an encoder–decoder architecture for the inverse design of sound-absorbing materials. The model used four structural parameters of the Helmholtz resonator to achieve inverse prediction. He et al. [34] applied reinforcement learning and tandem neural network architectures to the inverse design of the topological structures of phononic crystals. Xiao et al. [35] combined nine Helmholtz resonators into a 3 × 3 composite unit and used the neck diameter and length of each resonator as design variables. The model performed inverse design of the sound absorption curve and analyzed the effect of the number of resonators on design accuracy. Cho and Langfeld [36] proposed a scaling analysis method for the sound transmission loss of thin plate-type acoustic metamaterials, establishing relationships between the acoustic responses of geometrically scaled structures. Their scaling laws, derived from the thin-plate governing equation and validated by finite element simulations, provide an effective tool for simplifying design and facilitating scaled experiments.

Table 1 presents the structural configurations and sound absorption bandwidths of different acoustic metamaterials.

However, complex structures and numerous design parameters increase the difficulty of the design process. Traditional methods often depend on extensive parameter scanning and trial-and-error procedures, which are time-consuming and require high levels of expertise. Neural networks, as an important branch of machine learning, can simulate the connections and interactions between neurons in the human brain. By constructing complex models through multiple nodes and interconnections, they exhibit strong learning ability and adaptability. These characteristics make neural networks a promising tool for the design and optimization of acoustic metamaterials.

## 2. Low-Frequency Sound Insulation Characteristics of Thin-Plate Metamaterials

### 2.1. Theoretical Analysis of Sound Insulation of Thin Plate Metamaterials

**Figure 1:**
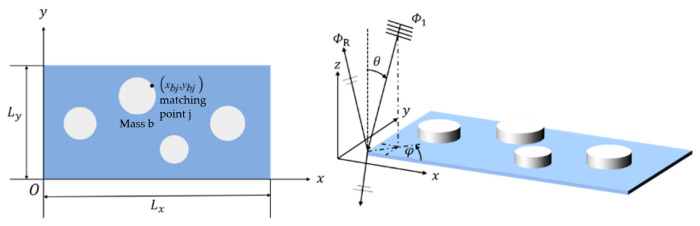
Thin-sheet metamaterials.

Figure 1 illustrates a rectangular thin-plate metamaterial with multiple cylindrical masses attached. The lower left corner of the plate is defined as the coordinate origin “O.” The two edges intersecting at point “O” represent the positive directions of the *x*- and *y*-axes. The plate is fully fixed along its boundaries, and several cylindrical masses are distributed on its upper surface. Let the lateral displacement of the plate be denoted by *w(x, y, t)*, the side lengths by *L_x_* and *L_γ_*, the thickness by *h*, and the density by *ρ_s_*. The Young’s modulus and Poisson’s ratio of the plate are represented by *E* and *ν*, respectively. Under acoustic excitation, the vibration of the plate can be expressed as [37]:

(1) $$D\nabla^{4}w\left(x,y,t\right)+\rho_{s}h\frac{\partial^{2}w\left(x,y,t\right)}{\partial t^{2}}\;=\;i\omega \rho_{0}(\Phi_{1}-\Phi_{2})+\sum_{b=1}^{N_{m}}\sum_{j=1}^{J_{b}}F_{bj}\delta (x-x_{bj})\delta (y-y_{bj})$$

Here, $D=\frac{E(1+i\eta )h^{3}}{12(1-\nu^{2})}$ represents the complex bending stiffness of the thin plate, where η is the damping loss factor. The operator $\nabla^{4}={\left(\frac{\partial^{2}}{\partial x^{2}}+\frac{\partial^{2}}{\partial y^{2}}\right)}^{2}$, and $i=\sqrt{-1}$. The term $\rho_{0}$ denotes the air density, $\Phi_{1}$ and $\Phi_{2}$ are the acoustic velocity potentials of the incident and transmitted sound fields, respectively, and $N_{m}$ is the total number of cylindrical masses attached to the plate. In this model, the point matching method is used to convert the force exerted by each mass block on the plate into a set of discrete point forces distributed along the interface between the mass block and the plate. $J_{b}$ is the total number of matching points on the b-th mass block, and $F_{bj}$ represents the force applied by the j-th matching point on the b-th mass block. δ is the Dirac delta function, and $x_{bj}$ and $y_{bj}$ denote the x- and y-coordinates of the j-th matching point on the b-th mass block.

For obliquely incident plane simple harmonic waves, the expression of the acoustic velocity potential is:

(2) $$\Phi_{1}=\Phi_{\mathrm{inc}}+\Phi_{\mathrm{ref}}$$

The expressions for $\Phi_{\mathrm{inc}}\;$ and $\Phi_{\mathrm{ref}}$ are

(3) $$\begin{array}{l} \Phi_{\mathrm{inc}}=\Phi_{1}e^{-i(k_{x}x+k_{y}y+k_{z}z-\omega t)}\\ \Phi_{\mathrm{ref}}=\Phi_{R}e^{-i(-k_{x}x-k_{y}y-k_{z}z-\omega t)} \end{array}$$

Here, $\Phi_{1}$ denotes the amplitude of the acoustic velocity potential of the incident wave, and $\Phi_{R}$ denotes that of the reflected wave. In the spherical coordinate system shown in the right panel of Figure 1, ω is the angular frequency, θ is the incident pitch angle, and ϕ is the incident azimuth angle.

$k_{x}$, $k_{y}$ and $k_{z}$ are the wave number components of the incident sound wave in the x axis, y axis and z axis directions, respectively,

(4) $$\left\{\begin{array}{l} k_{x}=k_{0}\;\cos\varphi e\sin\theta \\ k_{y}=k_{0}\;\sin\varphi \sin\theta \\ k_{z}=k_{0}\;\cos\theta \end{array}\right.$$

where $k_{0}=\omega /c_{0}$ is the wave number and $c_{0}$ is the speed of sound wave propagation.

When a thin plate structure is excited by periodic acoustic waves, under fixed constraint boundary conditions, the normal vibration displacement of the plate can be described as

(5) $$w(x,y,t)=\sum_{m=1}^{\infty }\sum_{n=1}^{\infty }\Psi_{mn}(x,y)p_{mn}(t)$$

where $\Psi_{\mathrm{mn}}(x,y)$ is the modal shape function of the thin plate and $P_{mn}(t)$ is the corresponding modal displacement. In the case of a thin plate clamped on all four sides, they can be expressed as

(6) $$\Psi_{mn}(x,y)=\left[1-\cos\left(\frac{2m\pi x}{L_{x}}\right)\right]\left[1-\cos\left(\frac{2n\pi y}{L_{y}}\right)\right]$$

(7) $$p_{mn}\left(t\right)=\alpha_{mn}e^{i\omega t}$$

where $\alpha_{\mathrm{mn}}$ are the coefficients of the plate modal displacements.

The velocity continuity condition needs to be met on the coupling surface where the air and the thin plate contact each other.

(8) $$z=0:-\frac{\partial \Phi_{1}}{\partial z}=i\omega w,\;-\frac{\partial \Phi_{2}}{\partial z}=i\omega w$$

The velocity potential functions of the incident and radiated sound fields approaching the thin plate can be expressed as [38]

(9) $$\Phi_{1}=\sum_{m}^{\infty }\sum_{n}^{\infty }I_{mn}\Psi_{mn}(x,y)e^{-i(k_{z}z-\omega t)}+\sum_{m}^{\infty }\sum_{n}^{\infty }\beta_{mn}\Psi_{mn}(x,y)e^{-i(-k_{z}z-\omega t)}$$

(10) $$\Phi_{2}=\sum_{m}^{\infty }\sum_{n}^{\infty }\xi_{mn}\Psi_{mn}(x,y)e^{-i(k_{z}z-\omega t)}$$

where $I_{\mathrm{mn}}$ is the velocity potential amplitude coefficient of the incident sound wave, $\beta_{\mathrm{mn}}$ is the velocity potential amplitude coefficient of the reflected sound wave, and $\xi_{mn}$ is the velocity potential amplitude coefficient of the transmitted sound wave. Substituting Equations (9) and (10) into Equation (8), we obtain the following relationship:

(11) $$\beta_{mn}=I_{mn}-\frac{\omega \alpha_{mn}}{k_{z}}$$

(12) $$\xi_{mn}=\frac{\omega \alpha_{mn}}{k_{z}}$$

(13) $$I_{mn}=\int_{0}^{L_{x}}\int_{0}^{L_{y}}\Phi_{1}e^{-i\left(k_{x}x+k_{y}y\right)}\Psi_{mn}\left(x,y\right)dxdy$$

The relationship between sound pressure and sound velocity potential is as follows

(14) $$\left\{\begin{array}{l} p_{1}=\rho_{0}\frac{\partial \Phi_{1}}{\partial t}=i\omega \rho_{0}\Phi_{1}\\ p_{2}=\rho_{0}\frac{\partial \Phi_{2}}{\partial t}=i\omega \rho_{0}\Phi_{2} \end{array}\right.$$

When the thin plate is subjected to an oblique incident sound wave, the pressure difference on the plate is

(15) $$p_{e}(x,y,t)=i\omega \rho_{o}(\Phi_{1}-\Phi_{2})$$

In this theory, the point matching method is applied to transform the force between the cylindrical mass block and the thin plate into a set of point forces on the contact surface boundary. The mass of the cylindrical block is then evenly distributed to each matching point, that is,

(16) $$m_{bj}=\frac{m_{b}}{J_{b}}$$

(17) $$m_{b}=\rho_{m}\pi r_{b}^{2}h_{m}$$

where $m_{bj}$ is the mass at the j th matching point of the b th mass block, and $m_{b}$ is the total mass of the b th mass block. All masses considered in this study have the same density and height. $\rho_{m}$ denotes the density of the mass block, $h_{m}$ denotes its height, and $r_{b}$ denotes the radius of the b th cylindrical mass block. The force acting on the j th matching point of the b th mass block can be expressed as

(18) $$F_{bj}=-m_{bj}\frac{\partial^{2}w\left(x_{bj},y_{bj},t\right)}{\partial t^{2}}$$

Substituting Formulas (5), (7), (16) and (17) into Formula (18) yields

(19) $$F_{bj}\;=\;\frac{\omega^{2}\rho_{m}\pi r_{b}^{2}h_{m}}{J_{b}}\sum_{m=1}^{\infty }\sum_{n=1}^{\infty }\Psi_{mn}\;(x_{bj},y_{bj})\alpha_{mn}e^{i\omega t}$$

A set of matching points on the contact surface between the mass block and the thin plate are considered to be uniformly distributed. The coordinates of the matching points can be expressed as

(20) $$\left\{\begin{array}{l} x_{bj}=x_{b}+r_{b}\cos\left(\frac{2\pi \left(j-1\right)}{J_{b}}\right)\\ y_{bj}=y_{b}+r_{b}\sin\left(\frac{2\pi \left(j-1\right)}{J_{b}}\right) \end{array}\right.$$

where $x_{b}$ and $y_{b}$ are the x and y coordinates of the center of mass of the b th cylindrical mass.

Multiply both sides of Formula (1) by the function $\Psi_{\mathrm{rs}}\left(x,y\right)$, and use the modal superposition method to perform a weighted integral of the shape function on the plate’s vibration control equation and set it to zero. Solving the integral equation yields

(21) $$\int_{0}^{L_{x}}\int_{0}^{L_{y}}\left[\begin{array}{l} D\nabla^{4}w\left(x,y,t\right)+\rho_{s}h\frac{\partial^{2}w\left(x,y,t\right)}{\partial t^{2}}-i\omega \rho_{0}(\Phi_{1}-\Phi_{2})\\ -\sum_{b=1}^{N_{m}}\sum_{j=1}^{J_{b}}F_{bj}\delta (x-x_{bj})\delta (y-y_{bj}) \end{array}\right]\Psi_{rs}\left(x,y\right)dxdy=0$$

Substituting Formulas (5)–(7), (9)–(12), and (19) into Formula (21) yields

(22) $$\begin{array}{l} 4D\pi^{4}L_{x}L_{y}\left\{\begin{array}{l} \left[3{\left(\frac{r}{L_{x}}\right)}^{4}+3{\left(\frac{s}{L_{y}}\right)}^{4}+2{\left(\frac{r}{L_{x}}\right)}^{2}{\left(\frac{s}{L_{y}}\right)}^{2}\right]\alpha_{rs}\\ +\sum_{n}^{\infty }2{\left(\frac{r}{L_{x}}\right)}^{4}\alpha_{rn}+\sum_{m}^{\infty }2{\left(\frac{s}{L_{y}}\right)}^{4}\alpha_{ms} \end{array}\right\}+\frac{9L_{x}L_{y}}{4}Q_{rs}\\ +\sum_{n}^{\infty }\frac{3L_{x}L_{y}}{2}Q_{rn}+\sum_{m}^{\infty }\frac{3L_{x}L_{y}}{2}Q_{ms}+\sum_{m}^{\infty }\sum_{n}^{\infty }L_{x}L_{y}Q_{mn}\\ -\omega^{2}\rho_{m}\pi h_{m}\sum_{b=1}^{N_{m}}\sum_{j=1}^{J_{b}}\left\{\frac{r_{b}^{2}}{J_{b}}\left[\sum_{m=1}^{\infty }\sum_{n=1}^{\infty }\Psi_{mn}\left(x_{bj},y_{bj}\right)\alpha_{mn}\right]\Psi_{rs}\left(x_{bj},y_{bj}\right)\right\}\\ =2j\omega \rho_{0}I_{rs},\;\;\;\;\;z=0\left(r\neq m,s\neq n\right) \end{array}$$

where

(23) $$Q_{mn}=-\rho_{s}h\omega^{2}\alpha_{mn}+i\omega \rho_{0}\frac{2\omega }{k_{z}}\alpha_{mn}$$

Equations (22) and (23) are combined to form an infinite-dimensional algebraic system with the undetermined coefficients $\alpha_{rs}$. For numerical computation, the summation indices r and s are truncated to the ranges 1≪r≪M and 1≪s≪N. Under this constraint, the original problem is converted into a finite-dimensional algebraic system with MN equations, which can be expressed in matrix form.

(24) $$\left\{{\left[M_{rs}\right]}_{MN\times MN}-\sum_{b}^{N_{m}}\sum_{j}^{J_{b}}{\left[R_{bj}\right]}_{MN\times MN}\right\}{\left\{a_{rs}\right\}}_{MN\times 1}={\left\{F_{rs}\right\}}_{MN\times 1}$$

The relevant expressions in the formula are as follows

The unknown coefficient of the plate’s vibration displacement can be expressed as

(25) $$\left\{a_{rs}\right\}={\left[\alpha_{11}\cdots \alpha_{M1}\;\alpha_{12}\cdots \alpha_{M2}\cdots \alpha_{MN}\right]}_{MN\times 1}^{T}$$

The generalized force on the right side of Equation (24) is

(26) $$\left\{F_{rs}\right\}=2i\omega \rho_{0}{\left[I_{11}\cdots I_{M1}\;I_{12}\cdots I_{M2}\cdots I_{MN}\right]}_{MN\times 1}^{T}$$

We define a series of sub-matrices as

(27) $$\lambda_{rs}^{*1}=3{\left(\frac{r}{L_{x}}\right)}^{4}+3{\left(\frac{s}{L_{y}}\right)}^{+}+2{\left(\frac{r}{L_{x}}\right)}^{2}{\left(\frac{s}{L_{y}}\right)}^{2}$$

(28) $$\Delta_{1}^{*1}=diag{\left[\lambda_{11}^{*1}\;\ldots \;\lambda_{M1}^{*1}\;\;\lambda_{12}^{*1}\;\ldots \cdot \;\lambda_{M2}^{*1}\;\;\ldots \cdot \;\lambda_{MN}^{*1}\right]}_{MN\times MN}$$

(29) $$\lambda_{1,s}^{*2}=\frac{2s^{4}}{L_{y}^{4}}{\left[\begin{array}{ccccc} 0 & 1 & 1 & \cdots & 1\\ 1 & 0 & 1 & \cdots & 1\\ 1 & 1 & 0 & \cdots & 1\\ \vdots & \vdots & \vdots & & \vdots \\ 1 & 1 & 1 & \cdots & 0 \end{array}\right]}_{M\times N}$$

(30) $$\Delta_{1}^{*2}=diag{\left[\lambda_{1,1}^{*2}\;\lambda_{1,2}^{*2}\;\ldots \;\lambda_{1,N}^{*2}\right]}_{MN\times MN}$$

(31) $$\lambda_{1}^{*3}=\frac{2}{L_{x}^{4}}diag{\left[1^{4}\;\;\;\;2^{4}\;\;\;\cdots \;\;\;M^{4}\right]}_{M\times N}$$

(32) $$\Delta_{1}^{*3}={\left[\begin{array}{ccccc} 0 & \lambda_{1}^{*3} & \lambda_{1}^{*3} & \cdots & \lambda_{1}^{*3}\\ \lambda_{1}^{*3} & 0 & \lambda_{1}^{*3} & \cdots & \lambda_{1}^{*3}\\ \lambda_{1}^{*3} & \lambda_{1}^{*3} & 0 & \cdots & \lambda_{1}^{*3}\\ \vdots & \vdots & \vdots & & \vdots \\ \lambda_{1}^{*3} & \lambda_{1}^{*3} & \lambda_{1}^{*3} & \cdots & 0 \end{array}\right]}_{MN\times MN}$$

(33) $$\Delta_{2}^{*1}=\frac{9L_{x}L_{y}}{4}diag{\left[\begin{array}{cccc} 1 & 1 & \cdots & 1 \end{array}\right]}_{MN\times MN}$$

(34) $$\lambda_{2}^{*2}=\frac{3L_{x}L_{y}}{2}{\left[\begin{array}{ccccc} 0 & 1 & 1 & \cdots & 1\\ 1 & 0 & 1 & \cdots & 1\\ 1 & 1 & 0 & \cdots & 1\\ \vdots & \vdots & \vdots & & \vdots \\ 1 & 1 & 1 & \cdots & 0 \end{array}\right]}_{M\times N}$$

(35) $$\Delta_{2}^{*2}=diag{\left[\begin{array}{cccc} \lambda_{2}^{*2} & \lambda_{2}^{*2} & \cdots & \lambda_{2}^{*2} \end{array}\right]}_{MN\times MN}$$

(36) $$\lambda_{2}^{*3}=\frac{3L_{x}L_{y}}{2}diag{\left[\begin{array}{cccc} 1 & 1 & \cdots & 1 \end{array}\right]}_{M\times N}$$

(37) $$\Delta_{2}^{*3}={\left[\begin{array}{ccccc} 0 & \lambda_{2}^{*3} & \lambda_{2}^{*3} & \cdots & \lambda_{2}^{*3}\\ \lambda_{2}^{*3} & 0 & \lambda_{2}^{*3} & \cdots & \lambda_{2}^{*3}\\ \lambda_{2}^{*3} & \lambda_{2}^{*3} & 0 & \cdots & \lambda_{2}^{*3}\\ \vdots & \vdots & \vdots & & \vdots \\ \lambda_{2}^{*3} & \lambda_{2}^{*3} & \lambda_{2}^{*3} & \cdots & 0 \end{array}\right]}_{MN\times MN}$$

(38) $$\lambda_{2}^{*4}=L_{x}L_{y}{\left[\begin{array}{ccccc} 0 & 1 & 1 & \cdots & 1\\ 1 & 0 & 1 & \cdots & 1\\ 1 & 1 & 0 & \cdots & 1\\ \vdots & \vdots & \vdots & & \vdots \\ 1 & 1 & 1 & \cdots & 0 \end{array}\right]}_{M\times N}$$

(39) $$\Delta_{2}^{*4}={\left[\begin{array}{ccccc} 0 & \lambda_{2}^{*4} & \lambda_{2}^{*4} & \cdots & \lambda_{2}^{*4}\\ \lambda_{2}^{*4} & 0 & \lambda_{2}^{*4} & \cdots & \lambda_{2}^{*4}\\ \lambda_{2}^{*4} & \lambda_{2}^{*4} & 0 & \cdots & \lambda_{2}^{*4}\\ \vdots & \vdots & \vdots & & \vdots \\ \lambda_{2}^{*4} & \lambda_{2}^{*4} & \lambda_{2}^{*4} & \cdots & 0 \end{array}\right]}_{MN\times MN}$$

(40) $$\lambda_{3,rs}^{j}=\Psi_{rs}(x_{j},y_{j})$$

(41) $$L_{bj}={\left[\begin{array}{cccccccc} \lambda_{3,11}^{bj} & \cdots & \lambda_{3,M1}^{bj} & \lambda_{3,12}^{bj} & \cdots & \lambda_{3,M2}^{bj} & \cdots & \lambda_{3,MN}^{bj} \end{array}\right]}_{1\times MN}$$

According to the above definition of submatrix, we can get

(42) $${\left[M_{rs}\right]}_{MN\times MN}=4D\pi^{4}L_{x}L_{y}(\Delta_{1}^{*1}+\Delta_{1}^{*2}+\Delta_{1}^{*3})-(\rho_{s}h\omega^{2}-i\omega \rho_{0}\frac{2\omega }{k_{z}})(\Delta_{2}^{*1}+\Delta_{2}^{*2}+\Delta_{2}^{*3}+\Delta_{2}^{*4})$$

(43) $${\left[R_{bj}\right]}_{MN\times MN}=\frac{\omega^{2}\rho_{m}\pi r_{b}^{2}h_{m}}{J_{b}}L_{bj}^{T}L_{bj}$$

After obtaining the modal coefficient $\alpha_{rs}$ from Equation (24) and substituting it into Equation (5), the vibration displacement, vibration velocity, and acoustic velocity potential of the metamaterial plate can be determined. The transmission coefficient of sound power depends on the incident wave’s pitch angle θ and azimuth angle ϕ. The sound power on both sides of the thin plate can be expressed as [39]

(44) Πi=12Re∬Apiνi*dA, i=1,2

The particle velocity in the sound field can be expressed in terms of sound pressure as $\nu_{i}=p_{i}/(\rho_{0}c_{0})$. In Equation (44), the superscript * denotes the complex conjugate. The transmission coefficient of the sound wave is

(45) $$\tau \left(\varphi ,\theta \right)=\frac{\Pi_{2}}{\Pi_{1}}$$

Taking the inverse of the transmission coefficient and expressing it in decibels gives the sound transmission loss

(46) $$STL=10\mathrm{lg}(\frac{1}{\tau (\varphi ,\theta )})$$

### 2.2. Simulation Verification of Sound Insulation of Thin Plate Metamaterials

#### 2.2.1. Geometric Model and Parameter Setting

**Figure 2:**
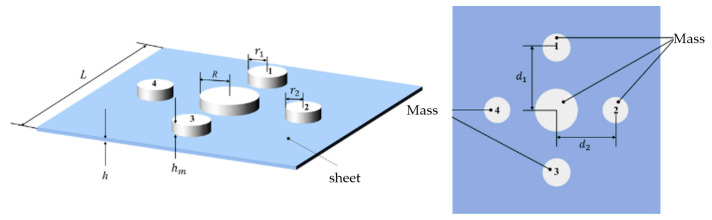
Designing thin-plate metamaterials.

In this study, a thin-plate metamaterial with multiple cylindrical masses is designed. The geometric model is shown in Figure 2. The metamaterial consists of a thin plate, one large mass, and four small masses. The thin plate has a square cross-section in the xy plane, with $L_{x}=L_{y}=L$. The large mass is positioned at the center of the thin plate, while the four small masses are arranged along the centerlines in the x and y directions. The radii and center-to-plate-center distances of mass blocks 1 and 3 are identical, as are those of mass blocks 2 and 4. All masses have the same height. The structural parameters of the metamaterial include the side length L and thickness h of the thin plate, the radius R of the large mass, the radii $r_{1}$ of mass blocks 1 and 3 and $r_{2}$ of mass blocks 2 and 4, and the distances $d_{1}$ and $d_{2}$ between the centers of mass of the small masses and the center of the thin plate.

This section presents a theoretical calculation of the sound insulation performance of the designed thin metamaterial. The number of truncated modes in each direction of the thin plate is set to M=N=140. Each of the four small masses has 40 matching points, while the central large mass has 80 matching points. The calculation assumes normal incidence of the sound wave (θ=0,ϕ=0) with an amplitude of $\Phi_{1}=1\;\mathrm{Pa}$. The damping loss factor of the thin plate is η=0.001, and the air is under standard atmospheric conditions, with a density of $\rho_{0}=1.225\;\mathrm{kg}/m^{3}$ and a sound speed of $c_{0}=340\;m/s$. The thin plate has an edge length of L=80 mm. The central large mass has a radius of R=10 mm, and the four small masses have radii $r_{1}=r_{2}=5\;mm$. The distances between the centers of the small masses and the plate center are $d_{1}=d_{2}=24\;mm$. The frequency range is selected from f=1 to 1000 Hz. Table 2 presents the material parameters of the thin plate and the mass block.

#### 2.2.2. Finite Element Simulation Settings

**Figure 3:**
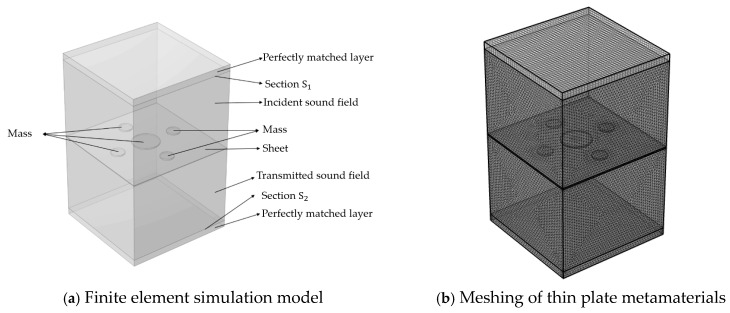
Finite element simulation model and meshing of thin plate metamaterials.

To validate the accuracy of the analytical model, the sound insulation results of the designed thin-plate metamaterial are compared with numerical simulation data. A finite element model of the metamaterial is constructed in COMSOL Multiphysics 6.0 using the acoustic-structure interaction module. The three-dimensional simulation model is shown in Figure 3. From top to bottom, the vertical components are: perfectly matched layer (PML), incident sound field, thin-plate metamaterial, transmitted sound field, and another PML. The incident sound field is the region where the sound wave is generated. After passing through the metamaterial, the wave propagates to the transmitted sound field. The PMLs on the top and bottom are used to simulate an infinite sound field, ensuring that reflected and transmitted waves propagate in one direction and decay gradually to zero. The contact surface between the upper PML and the incident sound field is defined as section $S_{1}$, and that between the lower PML and the transmitted sound field is defined as section $S_{2}$.

The thin plate is fixed along all edges. The lateral boundaries of the perfectly matched layers, incident sound field, and transmitted sound field are defined as rigid acoustic boundaries. The incident sound field is set to a background pressure of $P_{in}=1\;\mathrm{Pa}$, with the sound wave incident vertically. The sound insulation of the thin-plate metamaterial is calculated as follows:

(47) $$STL=10\log_{10}(\frac{W_{\mathrm{in}}}{W_{\mathrm{out}}})$$

where $W_{\mathrm{in}}$ and $W_{\mathrm{out}}$ are the incident sound power and the transmitted sound power, respectively, and the expression is

(48) $$W_{\mathrm{in}}=\int_{S_{1}}\frac{P_{\mathrm{in}}^{2}}{2\rho_{0}c_{0}}dS$$

(49) $$W_{\mathrm{out}}=\int_{S_{2}}\frac{P_{\mathrm{tr}}^{2}}{2\rho_{0}c_{0}}dS$$

#### 2.2.3. Verification and Analysis of Theoretical Models

**Figure 4:**
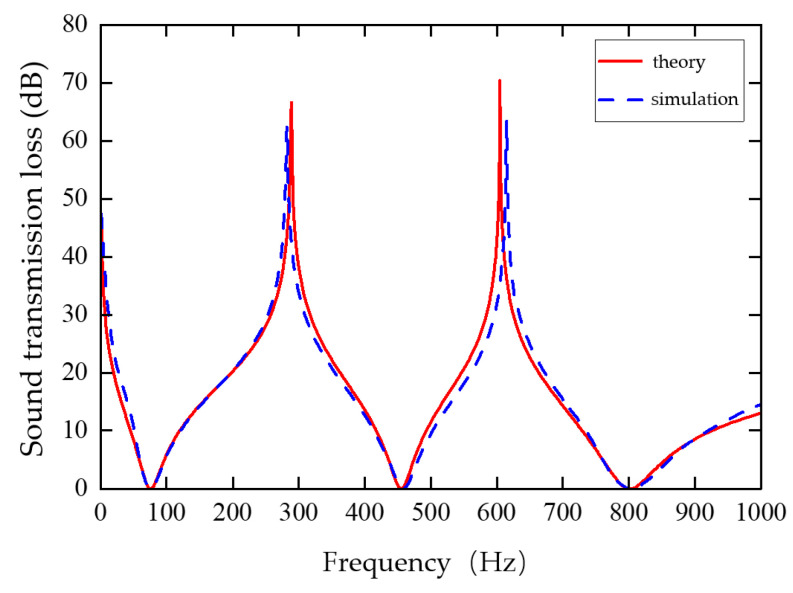
Comparison of theoretical results and simulation results.

**Figure 5:**
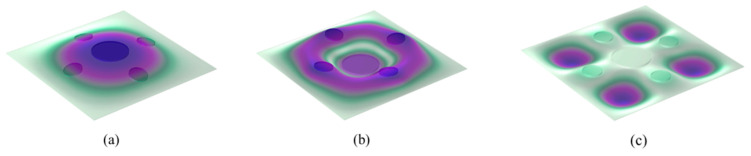
Eigenmodes of thin-plate metamaterial (**a**) 75.48 Hz (**b**) 459.61 Hz (**c**) 806.24 Hz.

**Figure 6:**
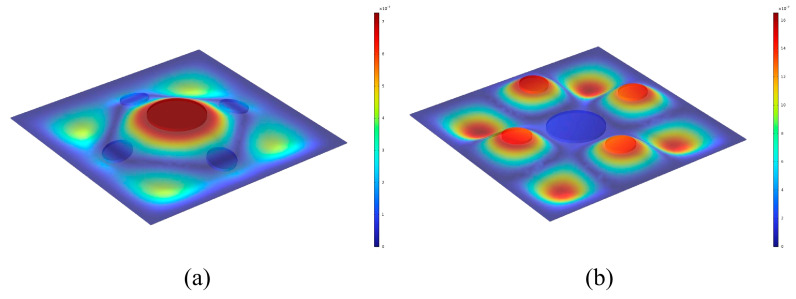
Surface vibration velocity of thin plate metamaterial at peak sound insulation frequency (**a**) 282 Hz (**b**) 615 Hz.

The theoretical and finite element simulation results are presented in Figure 4, showing good agreement. The calculated sound insulation valleys occur at 76 Hz, 455 Hz, and 801 Hz, corresponding to approximately 0 dB, while the sound insulation peaks occur at 289 Hz (66.6 dB) and 605 Hz (70.4 dB). The deviation between the theoretical and simulated frequencies for each peak and valley is less than 10 Hz, confirming the high accuracy of the theoretical model. Figure 5 illustrates the three eigenmodes at 75.48 Hz, 459.61 Hz, and 806.24 Hz, all of which are symmetrical. In the first-order mode, the large and small masses move in the same direction. In the second-order mode, they move in opposite directions. In the third-order mode, the masses remain essentially stationary, and only the four corners of the plate vibrate. Figure 6 shows the surface vibration velocity distribution at the sound insulation peaks. At the first peak frequency, the small mass blocks are nearly stationary, and the motion is mainly due to the large mass block and its adjacent plate area. At the second peak frequency, the large mass block is nearly stationary, and the vibration is primarily caused by the small mass blocks and their surrounding areas, which move in opposite directions.

#### 2.2.4. Sound Insulation Performance of Double-Layer Thin-Plate Acoustic Metamaterials

**Figure 7:**
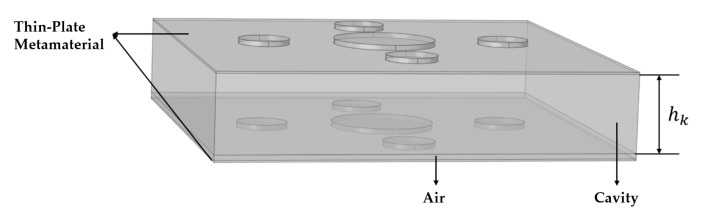
Double-Layer Thin-Plate Metamaterial.

Based on previous studies, stacking thin-plate metamaterials can generate multiple sound insulation peaks and effectively broaden the insulation bandwidth. In this section, a double-layer thin-plate metamaterial is investigated to examine the influence of stacking on sound insulation performance. The schematic of the double-layer structure is shown in Figure 7, which consists of two thin-plate metamaterial layers, referred to as the upper and lower layers.

**Figure 8:**
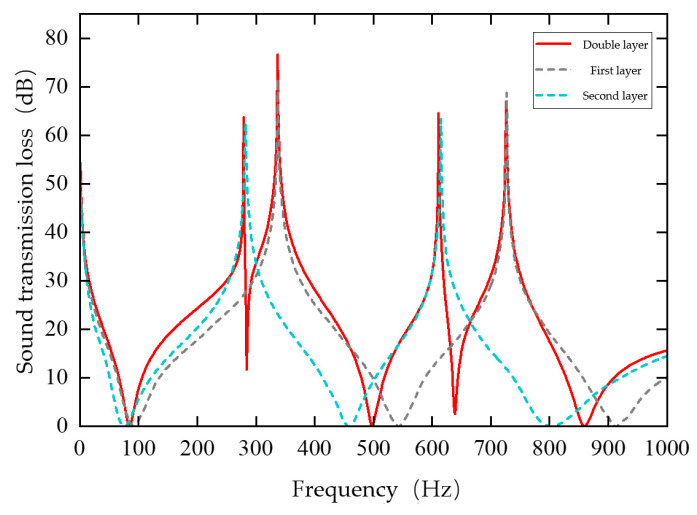
Sound Transmission Loss Curve of the Double-Layer Thin-Plate Metamaterial.

An air cavity is formed between the lower surface of the upper plate and the upper surface of the mass blocks of the lower plate, with a cavity height of 2 mm. The region between the upper surface of the lower plate and the cavity, excluding the mass blocks, is also filled with air. The structural parameters of the two layers are listed in Table 3. The two layers differ only in plate thickness, which is 0.35 mm for the upper layer and 0.40 mm for the lower layer, while all other parameters remain identical. Both plates are subjected to fixed boundary conditions along their edges. The remaining simulation settings are consistent with those used previously. Numerical simulations are carried out using COMSOL, and the corresponding sound insulation curves are shown in Figure 8.

As illustrated in Figure 8, the curves of the single-layer metamaterials with corresponding parameters are also provided as reference. The double-layer thin-plate metamaterial exhibits four sound insulation peaks and five valleys, which is two peaks and two valleys more than those of the single-layer structure. The sound insulation valleys occur at 84 Hz, 284 Hz, 498 Hz, 639 Hz, and 859 Hz. The four insulation peaks appear at 279 Hz, 337 Hz, 611 Hz, and 726 Hz, with maximum sound insulation levels of 63.7 dB, 76.6 dB, 64.6 dB, and 66.9 dB, respectively.

The first and third peaks correspond to the resonance peaks of the upper-layer metamaterial, while the second and fourth peaks are associated with those of the lower layer. Compared with the single-layer thin-plate metamaterial, the double-layer configuration produces more insulation peaks and achieves a significantly broader sound insulation bandwidth.

Based on the designed sound insulation frequency range of the thin-plate metamaterial and the sound insulation characteristics of the double-layer configuration, stacking four thin-plate metamaterial layers is expected to achieve favorable sound insulation performance over the frequency range of 1–1000 Hz. Therefore, a four-layer composite thin-plate metamaterial is selected for subsequent analysis.

## 3. Inverse Design of Single Cell Model of Multilayer Composite Thin Plate Metamaterial Based on Neural Network

### 3.1. Dataset Creation

The unit cell of the thin-plate acoustic metamaterial is selected based on the assumption of an infinite periodic arrangement in the in-plane directions. Each unit cell consists of a rectangular thin plate with periodically attached cylindrical mass blocks. For multilayer configurations, multiple identical or parameter-varied unit cells are stacked along the thickness direction. The geometric dimensions and material properties of the unit cell, including the plate thickness, unit-cell size, mass block radius, height, position, and material parameters, are summarized in Table 3. The vibroacoustic behavior of the structure is modeled using numerical simulations, assuming homogeneous, isotropic, and linearly elastic materials with small deformations. A normally incident plane acoustic wave is considered, and periodic boundary conditions are applied to neglect edge effects.

In order to ensure the reproducibility and reliability of the proposed neural-network-based inverse design framework, the neural network configuration, dataset scale, and validation strategy are briefly summarized here. A total of 30,000 samples were generated through coupled COMSOL–MATLAB numerical simulations of the multilayer thin-plate metamaterial unit cell. Among them, 24,000 samples were used for training and 6000 samples were reserved for independent testing. The neural network performance was evaluated using test-set errors, frequency-band error analysis, numerical reconstruction, and experimental verification using impedance tube measurements. Thin-plate acoustic metamaterials offer the advantages of being ultra-lightweight and having a programmable sound insulation frequency. Stacking multiple layers can expand the effective sound insulation bandwidth. However, increasing the number of layers also increases the number of structural parameters to be designed and requires consideration of inter-layer interactions, which complicates the design process. This chapter employs neural networks to provide new solutions for designing the structural parameters of multi-layer composite thin-plate metamaterial unit cells. First, the basic principles of neural networks are introduced. Then, a dataset for the multi-layer composite unit cell model is constructed and preprocessed using COMSOL Multiphysics 6.0 and MATLAB 2021b co-simulation. Next, a forward prediction network for the unit cell is built and trained. Subsequently, an inverse design network is constructed and trained with the aid of the pre-trained forward prediction network. Finally, a design example is generated using the trained inverse design network, and its accuracy is verified through impedance tube experiments.

**Figure 9:**
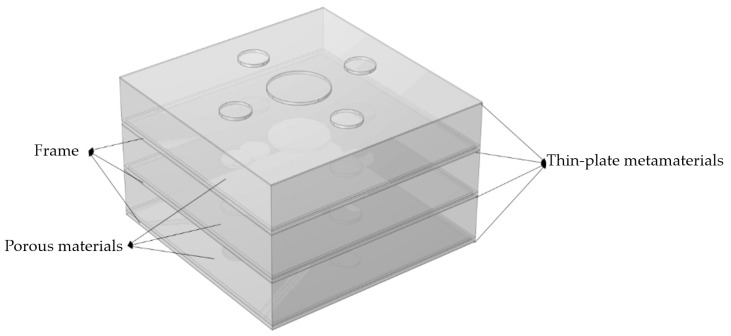
Single cell model of multilayer composite thin plate metamaterial.

Neural network training depends on the dataset, and its size directly affects the performance of the model. Therefore, constructing a reliable dataset is a critical step. The single-cell model of the multilayer composite thin-plate metamaterial used in this chapter is shown in Figure 9. The finite element model is essentially the same as that in Figure 3, except that the full metamaterial is replaced by a single-cell model. The figure description is omitted here. Stacking four layers of thin-plate metamaterials achieves effective sound insulation from 1 to 1000 Hz. This section focuses on the analysis of the four-layer composite thin-plate metamaterial. In this single-cell model, the upper surfaces of the thin plates in the lower three layers are attached to ring-shaped frames that primarily provide structural support. Each frame is covered with a porous material, which serves as a “peak-shaving and valley-filling” mechanism to improve the minimum sound insulation and facilitate neural network prediction. The medium between the lower surface of the porous material and the upper surface of the underlying thin-plate metamaterial, excluding the mass blocks and frames, is air. The material parameters of the thin plate, mass blocks, and frames are listed in Table 4.

The structural parameters of the thin-plate metamaterial include the plate side length L, plate thickness h, mass height $h_{m}$, large mass radius R, radii $r_{1}$ and $r_{2}$ of the four small masses, and the eccentricities $d_{1}$ and $d_{2}$ of the small masses. These parameters serve as the variables for neural network design. The plate side lengths of all layers are set to L. The frame has an outer side length of L, an inner side length of L−2mm, and a height of 1.5 mm. The porous material has a width of L and a height of 15 mm. No structural parameters are assigned to the frame or porous material. Except for the plate side length, all other structural parameters vary independently in each layer. The final structural parameters include the overall plate width L, and h, $h_{m}$, R, $r_{1}$, $r_{2}$, $d_{1}$, and $d_{2}$ for each layer. A total of 29 structural parameter variables are considered, and their value ranges are listed in Table 5. The ranges of $r_{1}$ and $r_{2}$ are identical, as are those of $d_{1}$ and $d_{2}$. Except for the minimum increment of h, which is 0.01 mm, the minimum increment for all other parameters is 0.1 mm.

In this section, a dataset is generated through joint simulation using COMSOL and MATLAB. First, global parameters are defined in COMSOL, and a four-layer composite thin-plate metamaterial unit cell model is constructed with assigned material properties. Pressure acoustics and solid mechanics are coupled for analysis. Fixed constraints are applied to the thin plate and frame boundaries, the mesh is generated, and the solution frequency is set from 1 to 1000 Hz with a 1 Hz step to calculate the sound insulation curve. Next, MATLAB calls the COMSOL interface to randomly sample structural parameters within the defined ranges and imports them into the model. The resulting sound insulation curves are returned to MATLAB and saved. This process is repeated to generate a total of 30,000 datasets, which are divided into training (24,000 sets) and test sets (6000 sets) at a 4:1 ratio.

### 3.2. Data Preprocessing

Data preprocessing is essential in neural network training, and normalization is one of the most common methods. It scales data with different units or magnitudes to a uniform range without changing the original characteristics. Normalization enables data comparison and computation on the same scale, accelerates training, improves model performance, and prevents gradient-related issues, ensuring algorithm stability and efficiency. It also simplifies parameter adjustment, enhances generalization ability, and helps the model converge to a global optimum. Common normalization methods include min–max normalization, which converts data to the range [0, 1]; Z-score normalization, which standardizes data to have a mean of 0 and a standard deviation of 1; L1 and L2 norm normalization, which adjust data based on absolute values or the Euclidean norm to handle sparsity or maintain directionality; and decimal scaling normalization, which rescales data by shifting the decimal point.

All structural parameter variables in this chapter have defined upper and lower limits; therefore, the min–max normalization method is applied to scale all parameters to the range [0, 1]. The normalization process ensures that each variable contributes proportionally during training and improves numerical stability. The specific formula is expressed as follows:

(50) $$x^{\prime }=\frac{x-x_{\min}}{x_{\max}-x_{\min}}$$

where *x* is the initial structural parameter, $x_{\min}$ and $x_{\max}$ are the minimum and maximum values in the initial structural parameter data, respectively, and $x^{\prime }$ represent the structural parameter data after normalization.

Since the sound insulation curve does not have a clear maximum and minimum value, the Z-Score normalization method is used to adjust the sound insulation data to a normal distribution with a mean of 0 and a standard deviation of 1. The conversion formula can be expressed as:

(51) $$x^{\prime }=\frac{x-\mu_{1}}{\sigma_{1}}$$

where *x* represents the raw sound insulation data, *μ*_1_ and *σ*_1_ denote the mean and standard deviation of all raw sound insulation data, respectively. The term *x*′ refers to the normalized sound insulation data. During neural network training, the test set data are unknown in advance. Therefore, the mean and standard deviation of the training set are used to normalize the test data. The calculated values are *μ*_1_ = 43.716 dB and *σ*_1_ = 20.017 dB.

### 3.3. Establishment and Training of Single-Cell Forward Prediction Network

**Figure 10:**
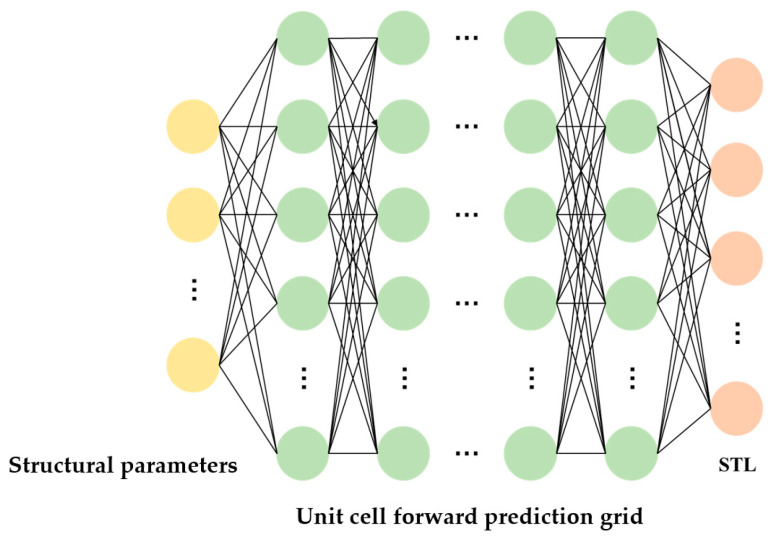
Schematic diagram of a single-cell forward prediction network.

The single-cell forward prediction network maps the structural parameters of a multi-layer composite thin-plate metamaterial unit cell to its corresponding sound insulation curve. When the structural parameters are provided, the corresponding sound insulation curve can be determined. This means that the input and output are in a one-to-one correspondence, and there is no problem of non-uniqueness. Therefore, a fully connected neural network is used to construct the single-cell forward prediction network, as shown in Figure 10. The input of the network consists of the structural parameters of the multi-layer composite thin-plate metamaterial unit cell. The metamaterial includes four layers of thin-plate metamaterials, with a total of 29 structural parameters. Accordingly, the input layer of the neural network contains 29 neurons. These parameters include the overall width *L* of the metamaterial, as well as *h_i_*, *h_mi_*, *R_i_*, *r*_1_*_i_*, *r*_2_*_i_*, *d*_1_*_i_*, and *d*_2_*_i_* for each thin-plate layer, where *i* = 1, 2, 3, and 4 represent the four layers from top to bottom. In the output layer, the sound insulation data are taken within the frequency range of 1–1000 Hz, with an interval of 1 Hz. Thus, the output layer contains 1000 neurons, each corresponding to the sound insulation value at 1 Hz, 2 Hz, 3 Hz, …, and 1000 Hz, respectively. The fitting relationship of the established single-cell forward prediction network can therefore be expressed as:

(52) $$\hat{Y}=F_{1}(X)$$

(53) $$\begin{array}{l} X=[L,h_{1},h_{m1},R_{1},r_{11},r_{21},d_{11},d_{21},h_{2},h_{m2}\cdots ,d_{14},d_{24}]\\ \hat{Y}=\left[{\hat{y}}_{1},{\hat{y}}_{2},{\hat{y}}_{3},\cdots ,{\hat{y}}_{1000}\right] \end{array}$$

where **X** denotes the structural parameter vector input to the neural network, *F*_1_ represents the single-cell forward prediction network, *ŷ_n_* (*n* = 1, 2, …, 1000) denotes the sound insulation value at *n* Hz predicted by the network, and *Ŷ* is the output sound insulation vector of the network. The frequency information is embedded in the position of the output sound insulation vector and does not need to be explicitly input or output in the neural network. The neurons can automatically learn this relationship during training.

To accelerate neural network training, the number of neurons in each hidden layer is set as an integer power of two. This configuration aligns with computer memory architecture and improves memory efficiency. Considering the effect of the number of layers and neurons on model accuracy, the single-cell forward prediction network is designed with four hidden layers. The first hidden layer contains 256 neurons, the second 512 neurons, the third 1024 neurons, and the fourth 256 neurons. Consequently, the network consists of six layers in total, including an input layer with 29 neurons, four hidden layers, and an output layer with 1000 neurons.

Predicting the sound insulation curve from the structural parameters of a single-cell model of a multilayer composite plate metamaterial is a nonlinear problem. An activation function is therefore required to enable the neural network to capture nonlinear relationships. The ReLU activation function is applied after each hidden layer of the single-cell forward prediction network. This function sets negative feature values to zero, enhances computational efficiency, and effectively prevents exploding and vanishing gradients.

During the training of the single-cell forward prediction network, the mean square error (MSE) function is employed as the loss function to measure the difference between the predicted and target values. The expression of the MSE is given as:

(54) $$MSE=\frac{1}{n}\sum_{i=1}^{n}{\left(\alpha_{i}-{\hat{\alpha }}_{i}\right)}^{2}$$

In the formula, *n* denotes the number of samples, *α_i_* represents the target value, and ${\hat{\alpha }}_{i}$ represents the predicted value. The MSE function calculates the mean of the squared differences between the target and predicted values for all samples. A smaller MSE value indicates lower prediction error and better model performance.

During the training of the single-cell forward prediction network, the Adam optimizer was employed to adjust the network gradients and ensure rapid convergence. Training was conducted for 500 epochs, with the training set reshuffled at the start of each epoch. The initial learning rate was set to 0.001, and at epoch 400, it was reduced to one-tenth of the original value. A higher learning rate in the early stages enables faster convergence toward the optimal solution, while a lower learning rate later reduces oscillations, allows finer parameter adjustments, and improves model accuracy. To enhance iteration efficiency and support convergence, mini-batch gradient descent was applied. The 24,000 datasets were divided into batches of 32 samples. During each iteration, only one batch was processed, and the model parameters were updated accordingly.

At this stage, the single-cell forward prediction network has been constructed, and the neural network hyperparameters have been configured. The next step is to load and process the dataset for training. The network training is carried out using the PyTorch 2.5.1 deep learning framework.

During neural network training, model performance is evaluated using the test set. Since the dataset has been normalized, the predicted sound insulation values must be denormalized to restore their original scale. This can be expressed by the following formula:

(55) $$\hat{x}={\hat{x}}^{\prime }\sigma +\mu$$

Among them,$\;{\hat{x}}^{\prime }$ is the predicted value of the neural network, and $\hat{x}$ is the denormalized value.

In order to evaluate the difference between the sound insulation predicted by the neural network and the target sound insulation, it is necessary to select a suitable evaluation standard. The evaluation formula can be expressed as:

(56) $$error=\frac{{\left\|\hat{Y}-Y\right\|}_{2}}{{\left\|Y\right\|}_{2}}\times 100\%$$

Here, ‖‖_2_ represents the 2-norm, $\hat{Y}$ is the neural network-predicted sound insulation vector, and **Y** is the target sound insulation vector. Note that normalization and denormalization can be considered steps in the neural network prediction operation, so we will not use other symbols to distinguish between the data before and after normalization or denormalization.

**Figure 11:**
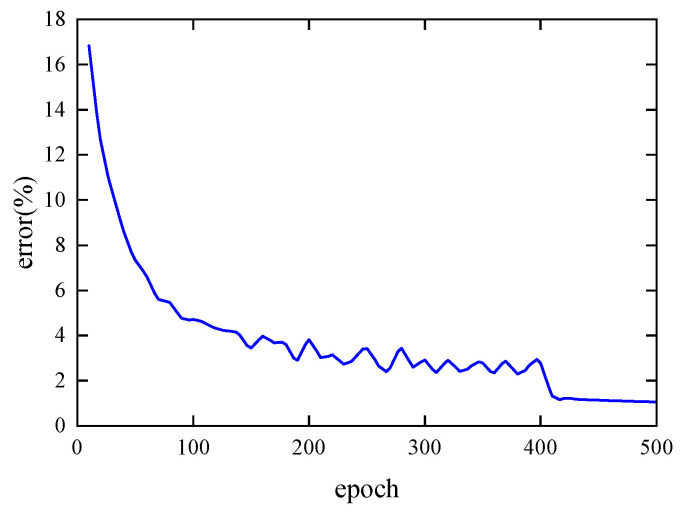
Error curve of the single-cell forward prediction network on the test set.

Figure 11 shows the error curve on the test set during the training of the single-cell forward prediction network. The error was calculated every 10 epochs. As shown, when the number of epochs is less than 100, the network error decreases rapidly with minimal fluctuation. Between 100 and 400 epochs, the error curve exhibits oscillations, but the overall error continues to decrease gradually. After 400 epochs, the reduced learning rate allows for finer parameter adjustments, leading to an initially rapid decrease in error followed by gradual convergence. At 500 epochs, the final error reaches 1.06%.

**Figure 12:**
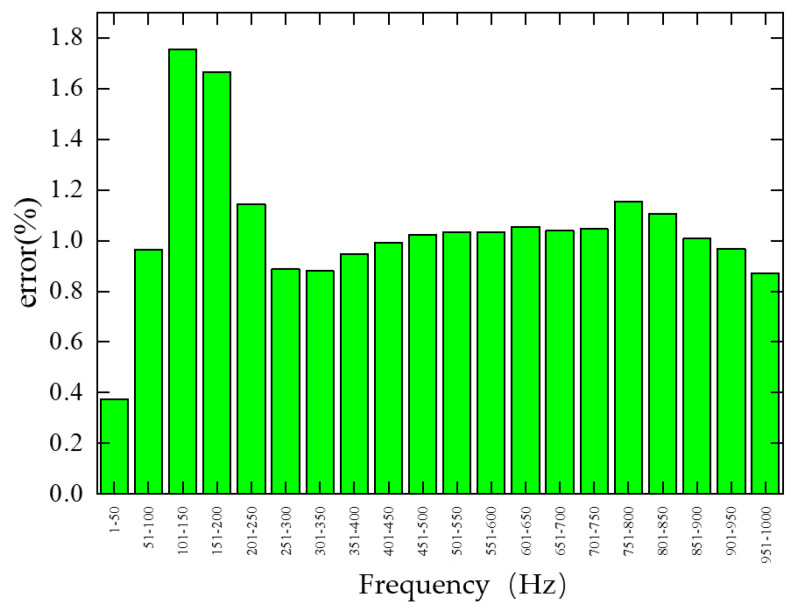
Frequency band error of the single-cell forward prediction network on the test set.

To visualize the frequency-dependent error of the trained single-cell forward prediction network on the test set, the error was calculated every 50 Hz within the 1–1000 Hz range, as shown in Figure 12. The network exhibits relatively large errors in the 101–150 Hz and 151–200 Hz bands, reaching 1.76% and 1.67%, respectively, indicating significant sensitivity to structural parameter variations in these regions. Errors are also notable in the 201–250 Hz and 751–800 Hz bands, at 1.14% and 1.16%, respectively. Conversely, smaller errors occur in the 251–300 Hz, 301–350 Hz, and 951–1000 Hz bands, at 0.89%, 0.88%, and 0.87%, respectively. In particular, the 1–50 Hz band shows the lowest error at 0.37%, suggesting minimal variation with structural parameter changes. Errors in the remaining frequency bands are close to the average test set error.

**Figure 13:**
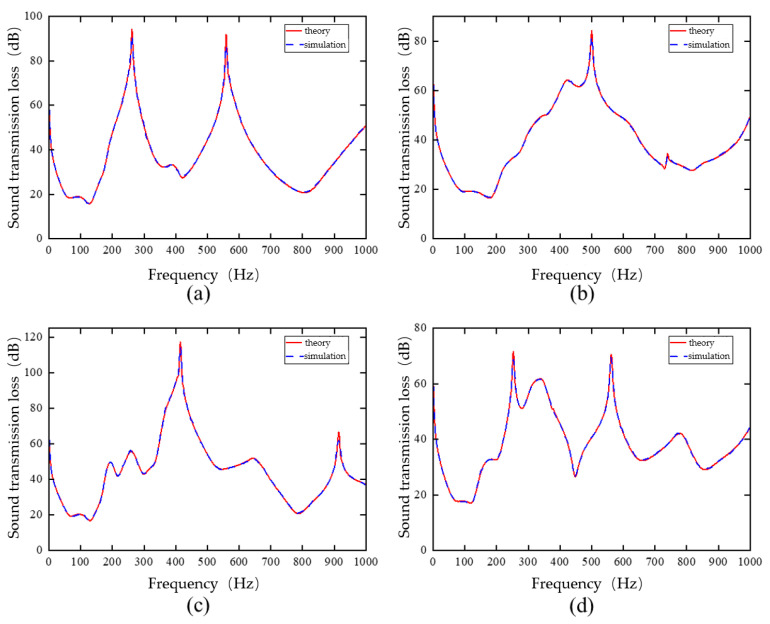
Simulation values and single-cell forward prediction network prediction values under different parameters: (**a**) Sample 1, (**b**) Sample 2, (**c**) Sample 3, (**d**) Sample 4.

To verify the accuracy of the trained single-cell forward prediction network, four sets of structural parameters were selected from the test set. These parameters were input into the network, and the predicted sound insulation curves were compared with the simulated curves. The predicted results were denormalized before comparison. The corresponding results are shown in Figure 13.

Figure 13 presents the four cases with different input parameter vectors. The red curves represent the simulated sound insulation, while the blue curves show the predictions from the neural network. The simulated and predicted curves exhibit similar magnitudes, trends, and shapes across all cases. Within the 1–1000 Hz frequency range, the predicted curves closely match the simulated results.

These results indicate that the trained single-cell forward prediction network can accurately predict the sound insulation curve for a given set of structural parameters. In other words, the network can effectively replace the simulation process that maps structural parameters to sound insulation curves. At this stage, the training of the single-cell forward prediction network is complete.

These results demonstrate that the forward prediction network can reliably replace time-consuming numerical simulations for mapping structural parameters to sound insulation curves.

### 3.4. Establishment and Training of Single-Cell Inverse Design Network

For the inverse design of a single-cell model of a multilayer composite plate metamaterial, the input is the target sound insulation curve, and the output is the 29 key structural parameters of the single-cell model. A straightforward approach is to directly construct a neural network that takes the target curve as input and outputs the designed structural parameters. However, inverse design often faces the problem of non-uniqueness: different structural parameters can produce very similar sound insulation curves. This non-uniqueness cannot be ignored in the inverse design of multilayer composite plate metamaterials.

During model training, non-uniqueness appears as similar input curves corresponding to significantly different outputs. This can slow the network convergence and increase model error. These challenges indicate that directly building a inverse design network using conventional methods is not feasible.

**Figure 14:**
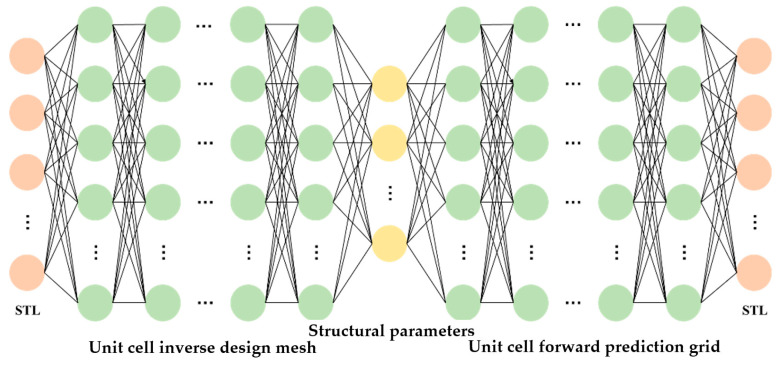
Schematic diagram of single-cell inverse design network training.

To overcome this issue, a trained single-cell forward prediction network is used to assist in training the inverse design network. The training scheme is shown in Figure 14. Specifically, the labeled sound insulation curve is input into the single-cell inverse design network to predict the structural parameters. These predicted parameters are then fed into the forward prediction network, whose parameters are frozen, to obtain the predicted sound insulation curve. The loss is calculated by comparing the predicted and labeled sound insulation curves. During backpropagation, only the parameters of the inverse design network are updated.

The single-cell inverse design network maps the sound insulation curve of a multilayer composite thin-plate metamaterial unit cell to its corresponding structural parameters. Its structure is roughly the inverse of the single-cell forward prediction network. The input layer contains 1000 neurons, corresponding to the sound insulation values at 1 Hz, 2 Hz, …, up to 1000 Hz. The output layer has 29 neurons, representing the structural parameters of the unit cell. These include the overall width *L* of the metamaterial, as well as *h_i_*, *h_mi_*, *R_i_*, *r*_1_*_i_*, *r*_2_*_i_*, *d*_1_*_i_*, and *d*_2_*_i_* for each thin-plate layer, where *i* = 1, 2, 3, 4 denotes the four layers counted from top to bottom. The training process of the single-cell inverse design network can be expressed as:

(57) $$\hat{X}=F_{2}\left(Y\right)$$

(58) $$\hat{Y}=F_{1}(\hat{X})$$

(59) $$\begin{array}{l} \hat{X}=[\hat{L},{\hat{h}}_{1},{\hat{h}}_{m1},{\hat{R}}_{1},{\hat{r}}_{11},{\hat{r}}_{21},{\hat{d}}_{11},{\hat{d}}_{21},{\hat{h}}_{2},{\hat{h}}_{m2}\cdots ,{\hat{d}}_{14},{\hat{d}}_{24}]\\ Y=\left[y_{1},y_{2},y_{3},\cdots ,y_{1000}\right] \end{array}$$

where *F_2_* denotes the single-cell inverse design network, and $\hat{L}$, ${\hat{h}}_{i}$, ${\hat{h}}_{mi}$, ${\hat{R}}_{i}$, ${\hat{r}}_{1i}$, ${\hat{r}}_{2i}$, ${\hat{d}}_{1i}$ and ${\hat{d}}_{2i}$ (*i* = 1, 2, 3, 4) are the structural parameters predicted by the network. $\hat{X}$ represents the predicted structural parameter vector, *y_n_* (*n* = 1, 2, …, 1000) denotes the target sound insulation value at *n* Hz, and *Y* is the target sound insulation vector input to the network. The meanings of other symbols are the same as in the previous section.

Training the single-cell inverse design network is challenging because it relies on a forward prediction network, making parameter adjustment susceptible to interference. To improve performance, a deeper network architecture is adopted. The network consists of an input layer with 1000 neurons, five hidden layers containing 1024, 256, 512, 512, and 128 neurons, respectively, and an output layer with 29 neurons, for a total of seven layers. Training uses only sound insulation curve data and employs an unsupervised learning approach. The network analyzes the input data to generate structural parameters, which are then passed through the forward prediction network to reconstruct the sound insulation curve.

The network used the ReLU activation function in the hidden layers and no activation function in the output layer. The mean square error (MSE) was used as the loss function, and the Adam optimizer was employed. Training was conducted for 1000 epochs with an initial learning rate of 0.0005, which was reduced to one-tenth after 800 epochs. Mini-batch gradient descent was applied with a batch size of 32 across a dataset of 24,000 samples. The test set error was evaluated every 10 epochs. The results show that the error decreased rapidly during the first 90 epochs, followed by slight fluctuations between 90 and 800 epochs, but the overall trend remained decreasing. After 800 epochs, the error stabilized and converged due to the reduced learning rate, ultimately reaching a convergence error of 2.27%.

**Figure 15:**
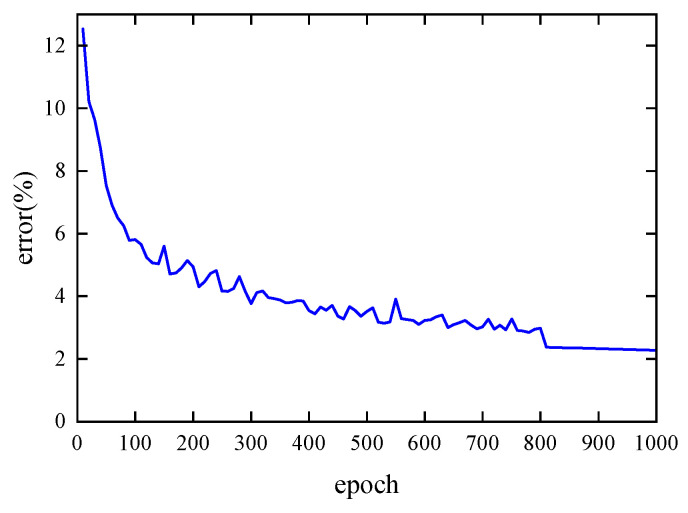
Error curve of the single-cell inverse design network on the test set.

As shown in Figure 15, the error curve of the single-cell inverse design network on the test set is presented.

**Figure 16:**
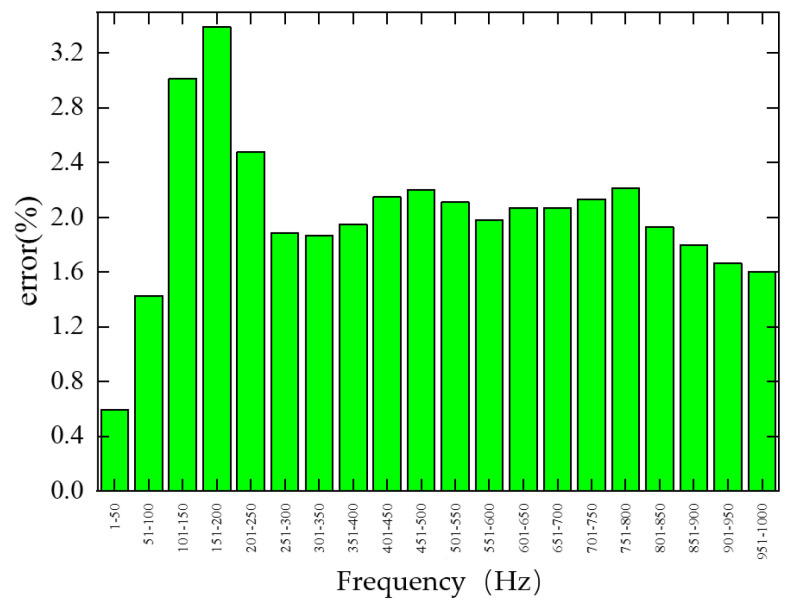
Frequency segmentation error of the single-cell inverse design network on the test set.

To visualize the frequency-dependent error of the trained single-cell inverse-design network on the test set, the error was calculated every 50 Hz within the 1–1000 Hz range, as shown in Figure 16. The network exhibits relatively large errors in the 101–150 Hz and 151–200 Hz bands, reaching 3.02% and 3.39%, respectively, indicating significant sensitivity to structural parameter variations in these regions. The 201–250 Hz band also shows a relatively high error of 2.48%. Smaller errors occur in the 51–100 Hz, 901–950 Hz, and 951–1000 Hz bands, at 1.43%, 1.66%, and 1.60%, respectively. The lowest error is observed in the 1–50 Hz band, at 0.59%, suggesting minimal variation with structural parameter changes. Errors in the remaining frequency bands are close to the average test set error.

**Figure 17:**
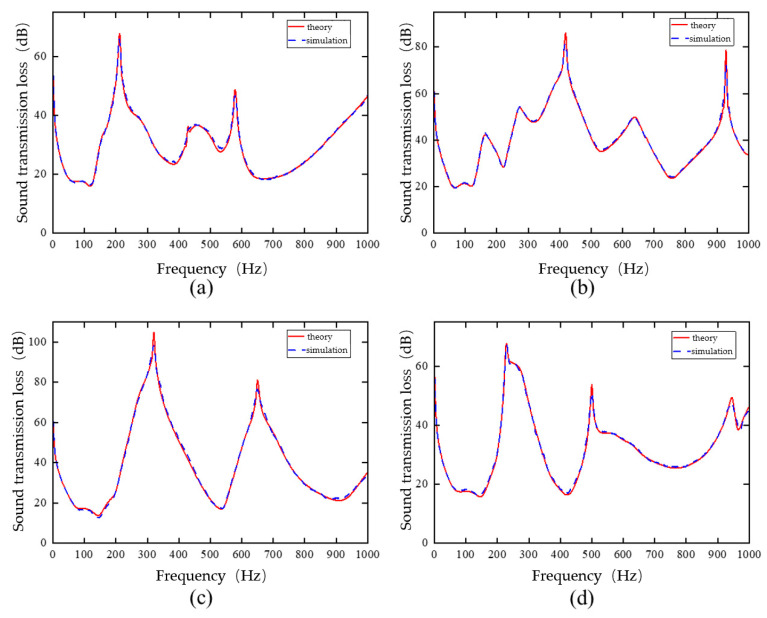
Simulation values and predicted values of the single-cell inverse design network under different parameters: (**a**) Sample 1, (**b**) Sample 2, (**c**) Sample 3, (**d**) Sample 4.

To evaluate the generalization performance of the single-cell inverse design network, four sets of sound insulation curves were selected from the test set. Since the network is trained using only sound insulation curve data, the predicted structural parameters were fed into the single-cell forward prediction network instead of using direct simulations to reconstruct the sound insulation curves. The predicted curves were then denormalized and compared with the corresponding simulated curves. The results are shown in Figure 17.

Figure 17 presents four examples with different input data. The blue curves represent the simulated sound insulation, while the red curves show the predicted curves from the network. The simulated and predicted curves are nearly identical across all four cases in terms of magnitude, variation trends, and overall shape. Within the 1–1000 Hz frequency range, the predicted curves closely match the simulated results. These results demonstrate that the neural network model for the inverse design of multilayer composite plate metamaterial single-cell models has been successfully constructed and trained.

The inverse-designed structures were further validated through impedance tube experiments, confirming the practical reliability of the neural-network-based inverse design approach.

## 4. Experimental Verification

### 4.1. Experimental Principle

**Figure 18:**
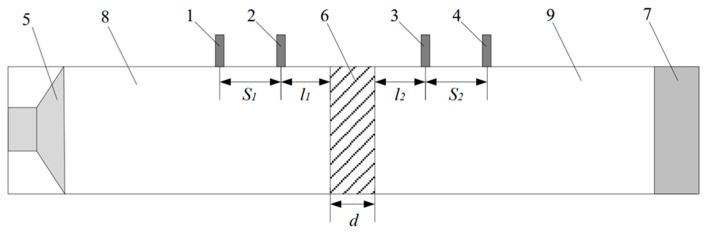
Impedance tube-four-sensor method test principle diagram.

Before presenting the experimental results, the measurement principle based on the impedance tube method is briefly introduced. Currently, the main experimental methods for measuring sound insulation are the soundproof box method, the impedance tube–four-sensor method, and the reverberation chamber–anechoic chamber method. The experiment in this section employs the impedance tube–four-sensor method. Relevant test standards can be found in GB/Z27764-2011 [40]. The test principle is illustrated in Figure 18. In the figure, 1–4 are acoustic sensors, 5 is a loudspeaker, 6 is the test sample, 7 is sound-absorbing material, 8 is the front tube, and 9 is the rear tube. During the experiment, the sound source in the front tube emits plane sound waves. After reflection and transmission through the test sample, some waves enter the rear tube and are absorbed by the sound-absorbing material.

The sound pressure in the front pipe is

(60) $$p(x)=p_{A}e^{jkx}+p_{B}e^{-jkx}$$

Similarly, the sound pressure in the rear pipe can be expressed as:

(61) $$p(x)=p_{C}e^{jkx}+p_{D}e^{-jkx}$$

We assume that the sound pressures measured by the four acoustic sensors in the experiment are $p_{1}$, $p_{2}$, $p_{3}$, and $p_{4}$, respectively. Substituting the measured sound pressures into Equations (60) and (61), we can solve for $p_{A}$, $p_{B}$, $p_{C}$, and $p_{D}$ to obtain:

(62) $$\begin{array}{l} p_{A}=\frac{1}{2j}\frac{p_{1}e^{-jkl_{1}}-p_{2}e^{-jk(l_{1}+S_{1})}}{\sin(kS_{1})}\\ p_{B}=\frac{1}{2j}\frac{p_{2}e^{jk(l_{1}+S_{1})}-p_{1}e^{jkl_{1}}}{\sin(kS_{1})}\\ p_{C}=\frac{1}{2j}\frac{p_{4}e^{jk(l_{2}-d)}-p_{3}e^{jk(l_{2}+S_{2}-d)}}{\sin(kS_{2})}\\ p_{D}=\frac{1}{2j}\frac{p_{3}e^{-jk(l_{2}+S_{2}-d)}-p_{4}e^{-jk(l_{2}-d)}}{\sin(kS_{2})} \end{array}$$

where $p_{A}$ and $p_{B}$ denote the amplitudes of the incident and reflected waves in the upstream tube, respectively, and $p_{C}$ and $p_{D}$ denote the amplitudes of the forward and backward propagating waves in the downstream tube.

Based on the solved wave amplitude coefficients $p_{A},\;p_{B},\;p_{C}$ and $p_{D}$ in Equation (62), the sound pressure and particle velocity on both sides of the specimen can be expressed as

(63) $$\left\{\begin{array}{l} p_{0}=p_{A}+p_{B}\\ u_{0}=\left(p_{A}-p_{B}\right)/\rho c \end{array}\right.\left\{\begin{array}{l} p_{d}=p_{C}e^{-jkd}+p_{D}e^{jkd}\\ u_{d}=\left(p_{C}e^{-jkd}-p_{D}e^{jkd}\right)/\rho c \end{array}\right.$$

where $p_{0}$ and $p_{d}$ are the sound pressures on the left and right sides of the test specimen, respectively, and $u_{0}$ and $u_{d}$ are the particle vibration velocities on the left and right sides of the test specimen, respectively. The relationship between them and the transfer matrix can be expressed as:

(64) $$\left[\begin{array}{c} p_{0}\\ u_{0} \end{array}\right]=\left[\begin{array}{cc} T_{11} & T_{12}\\ T_{21} & T_{22} \end{array}\right]\left[\begin{array}{c} p_{d}\\ u_{d} \end{array}\right]$$

The transfer matrix is derived and can be expressed as:

(65) $$T=\left[\begin{array}{cc} \frac{p_{d}u_{d}+p_{0}u_{0}}{p_{0}u_{d}+p_{d}u_{0}} & \frac{p_{0}^{2}-p_{d}^{2}}{p_{0}u_{d}+p_{0}u_{0}}\\ \frac{u_{0}^{2}-u_{d}^{2}}{p_{0}u_{d}+p_{0}u_{0}} & \frac{p_{d}u_{d}+p_{0}u_{0}}{p_{0}u_{d}+p_{d}u_{0}} \end{array}\right]$$

The calculated transmission coefficient is:

(66) $$\tau_{p}=\frac{2e^{jkd}}{T_{11}+T_{12}\;/\rho c+\rho cT_{21}+T_{22}}$$

The sound insulation is:

(67) $$STL=20\left|\frac{1}{\tau_{p}}\right|$$

### 4.2. Specimen Preparation and Experimental Platform

After training the single-cell inverse design network, the model is applied to inverse design the multilayer composite plate metamaterial unit cell. Since the network input is a sound insulation curve with 1000 discrete points, the target curve must first be generated. To improve design efficiency, an interpolation method is employed: key feature points of the target curve are selected, and their frequencies and sound insulation values are stored in a vector. Piecewise cubic Hermite interpolation is then used to calculate the sound insulation values at all 1000 frequency points, resulting in the complete target sound insulation curve.

The geometric parameters of the experimental samples are consistent with the inverse design results and are summarized in Table 6. All samples were fabricated in the laboratory to validate the proposed design method.

**Figure 19:**
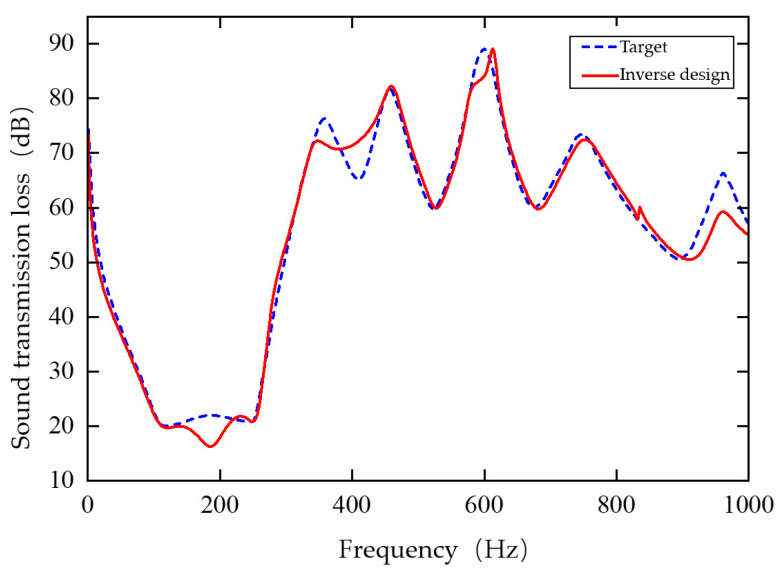
Comparison of the target sound insulation curve and the sound transmission loss curve designed by neural network.

Before inputting the target sound insulation curve into the single-cell inverse design network, it is normalized using the mean and variance of the training set. The structural parameters predicted by the network are then denormalized and input into the finite element model to obtain the inverse-designed sound insulation curve. Figure 19 compares the target and inverse-designed curves, and the predicted structural parameters are listed in Table 6. In the figure, the red curve represents the target sound insulation curve, while the blue curve represents the inverse design result. Although slight differences exist, the overall shapes and trends are consistent, demonstrating that the inverse design network can effectively predict the corresponding structural parameters from a target sound insulation curve.

**Figure 20:**
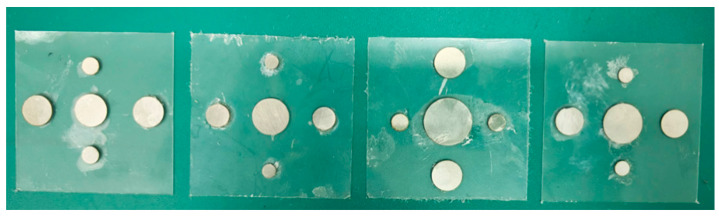
Thin plate metamaterial sample.

**Figure 21:**
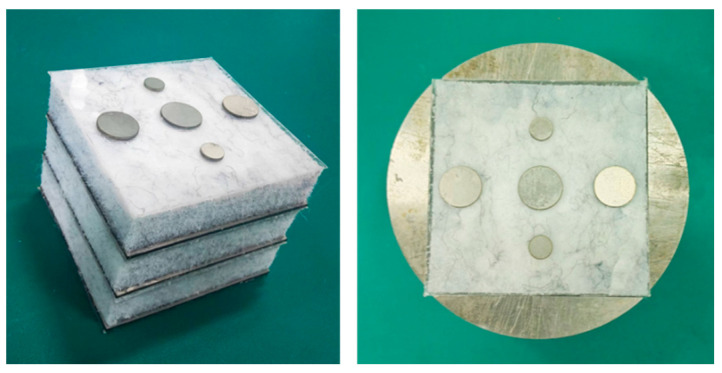
Experimental samples.

Figure 20 illustrates the structure of each layer of the multilayer composite thin-plate metamaterial unit cell, and the sample is shown in the left image of Figure 21. Since square impedance tubes matching the sample width were unavailable, circular impedance tubes were used for the experiment. The gaps were filled with steel blocks of the same thickness as the sample, as shown in the right image of Figure 21.

**Figure 22:**
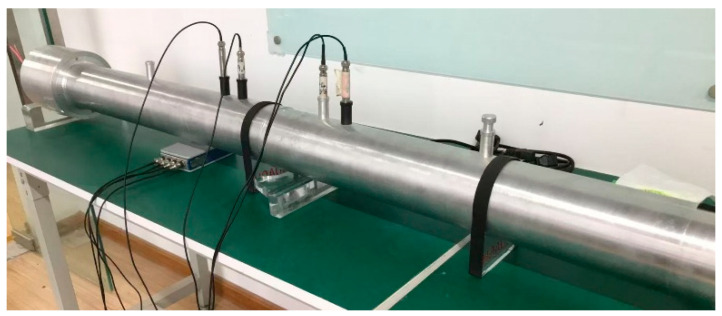
Experimental results analysis.

The experimental setup is shown in Figure 22. A BOACH-HW-CIT100(BOACH Acoustics, Suzhou, China) circular impedance tube was used. Because the sample was slightly wide, the four corners were polished before the experiment.

### 4.3. Results Analysis

**Figure 23:**
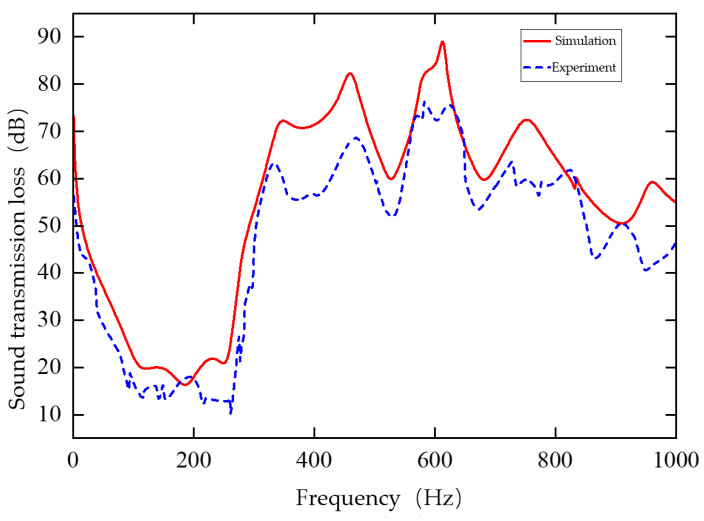
Comparison of experimental results with simulation results.

The comparison between the experimental and simulation results is shown in Figure 23. The experimental data are lower than the theoretical values, mainly due to inconsistencies in the boundary conditions between the specimen and the experimental setup. In the experiment, the edges of the sample may not be fully fixed or small air gaps may exist between the sample and the test tube. In contrast, the theoretical model assumes fully constrained or ideally coupled boundary conditions. This discrepancy has a significant effect at low frequencies, causing deviations in the curve trend. However, the overall trend of the experimental curve is consistent, verifying the agreement between the inverse-design results and the theoretical predictions.

## 5. Conclusions

This study investigates multilayer composite thin-plate acoustic metamaterials by integrating theoretical analysis, numerical simulation, neural-network-based inverse design, and experimental validation. A reliable theoretical model was established and verified through finite element simulations, demonstrating good agreement in predicting sound transmission loss characteristics in the low- and mid-frequency ranges.

The sound insulation performance of stacked thin-plate metamaterials was systematically analyzed. Results show that multilayer stacking generates additional insulation peaks and significantly broadens the effective sound insulation bandwidth compared with a single-layer configuration. Based on the analysis of the double-layer structure and the target frequency range of 1–1000 Hz, a four-layer composite thin-plate metamaterial was selected to achieve enhanced broadband sound insulation performance.

A neural-network-based inverse design framework was developed to efficiently design multilayer thin-plate metamaterial unit cells from target sound transmission loss curves. The inverse-designed structures exhibit sound insulation characteristics that are highly consistent with numerical predictions. Impedance tube experiments further validate the effectiveness and robustness of the proposed design approach.

Overall, the proposed multilayer thin-plate metamaterial combined with neural-network-based inverse design provides an efficient and flexible strategy for broadband sound insulation applications, offering strong potential for practical noise and vibration control engineering.

## Figures and Tables

**Table 1:** Structural configurations and sound absorption bandwidths of different acoustic metamaterials.

Acoustic Metamaterial	Reference	Structural Feature	Sound Absorption Bandwidth
thin-film acoustic metamaterial	Xiao et al. [4]	double-layer membrane acoustic metamaterial	70–200 Hz
	Xu et al. [5]	a free film, a supporting grating, and a back cavity	below 500 Hz
	Ciaburro et al. [13]	cork film as the membrane and thumbtacks and buttons as mass blocks	200–600 Hz
Thin-plate acoustic metamaterial	Langfeldt et al. [22]	various mass blocks and multilayer structures	100–400 Hz
	Wang et al. [24]	double-layer thin-plate metamaterial with a porous material	208–850 Hz

**Table 2:** Material parameters of thin plate and mass block.

Structure	Material	Density ρ (kg/m^3^)	Young’s Modulus	Poisson’s Ratio ν	Thickness h (mm)
sheet	PET	1432	3	0.39	0.35
Mass	steel	7860	210	0.3	1

**Table 3:** Structural Parameters of the Thin-Plate Metamaterial.

Thin-Plate Metamaterial	L (mm)	h (mm)	$h_{m}$ (mm)	R (mm)	$r_{1}$ (mm)	$r_{2}$ (mm)	$d_{1}$ (mm)	$d_{2}$ (mm)
First layer	80	0.40	1	10	5	5	24	24
Second laysr	80	0.35	1	10	5	5	24	24

**Table 4:** Material parameters of multilayer composite thin plate metamaterial.

Structure	Material	Density ρ (kg/m^3^)	Young’s Modulus	Poisson’s Ratio ν
sheet	PET	1432	3	0.39
Mass	steel	7860	210	0.3
frame	steel	7860	210	0.3

**Table 5:** Structural parameter value range.

Structural Parameters	Minimum Value (mm)	Maximum Value (mm)	Minimum Value Unit (mm)
*L*	70	90	0.1
*h*	0.25	0.45	0.01
$h_{m}$	0.5	1.5	0.1
*R*	7	13	0.1
$r_{1}$	3	7	0.1
$r_{2}$	3	7	0.1
$d_{1}$	20	27	0.1
$d_{2}$	20	27	0.1

**Table 6:** Structural parameters predicted by the unit cell inverse design network.

Metamaterials	L (mm)	h (mm)	$h_{m}$ (mm)	R (mm)	$r_{1}$ (mm)	$r_{2}$ (mm)	$d_{1}$ (mm)	$d_{2}$ (mm)
First layer	71.4	0.43	1.1	7.6	3.8	6.9	21.0	26.6
Second layer	71.4	0.34	1.0	8.1	3.1	5.2	23.8	22.4
Third layer	71.4	0.28	1.1	10.7	6.9	3.9	24.6	20.9
Fourth layer	71.4	0.45	0.7	9.0	3.4	6.3	20.4	24.6

## Data Availability

The original contributions presented in this study are included in the article. Further inquiries can be directed to the corresponding authors.
